# Cardiomyopathy in the Shadow of Fibrillary Glomerulonephritis: An Unusual Indirect Association

**DOI:** 10.7759/cureus.87879

**Published:** 2025-07-14

**Authors:** Satya Rijal, Maitri P Shah, Sai Sushrutha Mudupula Vemula, Prakash Khanal, Ayushma Duwadi

**Affiliations:** 1 Internal Medicine, University of Michigan Health-Sparrow Hospital, East Lansing, USA; 2 Internal Medicine, Michigan State University College of Human Medicine, East Lansing, USA

**Keywords:** endothelial injury, heavy proteinuria, interstitial fibrosis caused by the deposition of fibronectin and microfibrils in the vasculature, kidney disease and cardiac involvement and the success of chemotherapy, renin-angiotensin-aldosterone system

## Abstract

Fibrillary glomerulonephritis (FGN) is a rare and complex renal disease where the accumulation of non-amyloid microfibrils composed of polyclonal immunoglobulin G within the mesangium and glomerular capillaries results in structural and functional abnormalities in the kidney's filtering units. The direct link between FGN and cardiomyopathy is not well established. However, it may be caused secondarily by systemic inflammation, uremia, or other overlapping factors and comorbidities.

In our case, it was believed to be significantly influenced and worsened by various comorbid conditions, with hypertension being the most common cardiovascular risk factor in FGN itself, contributing to structural and functional cardiac impairment, including left ventricular hypertrophy and systolic dysfunction, rather than direct myocardial infiltration or damage by fibrillary deposit.

We have a case of a 60-year-old female who presented with a severe manifestation of FGN, associated with secondary cardiomyopathy and renal failure complications, including hypertension-related issues, nephrotic syndrome with significant proteinuria, peripheral edema, anasarca, and shortness of breath, initially leading to hospitalization. Her renal insufficiency subsequently progressed to end-stage renal disease, and the discovery of a nephrotic range of proteinuria led to a kidney biopsy, confirming the diagnosis of FGN. Her renal recovery remained poor due to persistent volume overload, which impeded renal recovery and which required the initiation of dialysis. The patient experienced a rare cardiac involvement related to underlying FGN, which could not be confirmed through a cardiac biopsy because she chose to refuse this invasive test.

The treatment with prednisone and rituximab targeted FGN, reducing proteinuria and improving renal function, which allowed for a decrease in hemodialysis and normalization of the erythrocyte sedimentation rate and C-reactive protein at 12 months. Cardiac function also improved after six months, with lower brain natriuretic peptide levels, increased left ventricular ejection fraction (LVEF), and reversal of ventricular remodeling on echocardiogram, suggesting that early immunologic intervention can reverse cardiomyopathy and prevent progression. Longer follow-up is recommended, as improved LVEF is associated with a better prognosis in heart failure. FGN typically affects the kidneys, making cardiac issues uncommon. Therefore, further research is needed to understand the prevalence and mechanisms of cardiac involvement in FGN for developing targeted therapies.

## Introduction

The underlying cause of fibrillary glomerulonephritis (FGN) is not fully understood, but genetic predisposition has been implicated in its development. Some studies postulated that human leukocyte antigen alleles and a combination of environmental and immune factors likely contribute to disease onset [1]. FGN-related cardiomyopathy involves indirect renal factors such as proteinuria, renin-angiotensin-aldosterone system (RAAS) activation, and interstitial fibrosis, and direct local cardiac expression of fibroblast growth factor 23 (FGF23) and RAAS components, suggesting a direct cardiac pathway that affects myocardial remodeling and fibrosis. Cardiac production of FGF23 can activate RAAS locally, increasing angiotensin II and aldosterone, which promote hypertrophy and remodeling [2]. 

FGN is identified in 0.5%-1% of native kidney biopsies [2]. The clinical presentation of FGN may vary from proteinuria (100%), nephrotic syndrome (38%), hematuria (52%), and hypertension (71%), which can progress to end-stage renal disease (ESRD) (44%) in a significant number of patients, mainly if not treated effectively. This applies to cohorts enrolled in observational chronic kidney disease (CKD) studies, mainly involving patients with advanced CKD (e.g., stage 4) who typically progress to ESRD over about five years. The time spent in these stages is usually short, often just months to a few years, with some patients progressing rapidly within five years [2-4].

It is often found coexisting with monoclonal gammopathy (10%) and autoimmune diseases (11%), including systemic lupus erythematosus (13%), rheumatoid arthritis (15%), and malignancy (23%) [5,6]. Studies are primarily derived from biopsy-based studies, which may overrepresent cases with comorbidities and suggest that the relationships between these underlying conditions and FGN may increase the likelihood of kidney injury due to prolonged inflammation and immune dysfunction [6,7]. Conditions that result in chronic inflammation, such as infections, hepatitis B and C (13%), or inflammatory disorders, may be a driving factor in the development of FGN, which involves the deposition of abnormal fibrils in the kidneys as part of the immune response [6]. While often idiopathic, it is essential to rule out infectious causes and autoimmune conditions when making a diagnosis [7].

The global age-standardized prevalence rate of CKD due to glomerulonephritis increased by 16% to 217.1 per 100,000 population between 1990 and 2019, indicating a substantial growth in the proportion of affected people over time. FGN is an uncommon renal disease with limited data on incidence and prevalence rate and a predilection for adult females ranging from 49 to 60 years [5,8,9].

While there is no universally accepted cure for FGN, some treatments, such as rituximab, a B-cell-depleting agent, have shown limited benefits in certain cases by reducing proteinuria and stabilizing kidney function. However, large-scale trials are lacking [2-4,6]. Plasma exchange (therapeutic plasma exchange) is generally ineffective for typical FGN because its mechanism involves fibrillary deposits mainly composed of immunoglobulins, which are not responsive to plasma removal. It may be considered only if a related condition, such as cryoglobulinemia or thrombotic microangiopathy, is present. Overall, treatment options are limited, and the role of plasma exchange in FGN is minimal unless a secondary, treatable condition exists [7-10].

## Case presentation

A 60-year-old woman with a history of smoking and hypertension presented to the ED with progressive shortness of breath, epigastric pain, and generalized body swelling (anasarca) and an increase in her weight of 10 lbs over a couple of days. Over the preceding three weeks, she experienced a persistent cough and worsening dyspnea, which gradually progressed in severity. In addition, she developed orthopnea, difficulty breathing when lying flat, and paroxysmal nocturnal dyspnea, characterized by sudden nighttime episodes of breathlessness that awakened her from sleep, indicating progressive fluid overload and cardiac compromise. She described right upper quadrant and epigastric pain for three weeks, with abdominal fullness or bloating. Importantly, this pain is usually vague, dull, or pressure-like in nature. The onset of epigastric pain alongside her respiratory symptoms raised concerns for cardiac ischemia or congestive heart failure with visceral congestion.

On physical examination, she had anasarca, manifesting as widespread edema potentially involving the extremities, face, abdomen, and genital area, reflecting a significant disruption of fluid homeostasis. 

In the ED, she was diagnosed with a hypertensive emergency, presenting with severely elevated blood pressure (BP) measuring 211/110 mmHg. Serum troponin was 300 ng/mL, brain natriuretic peptide (BNP) was 954 pg/mL, serum creatinine was 5.6 mg/dL (normal: 0.6-1.4 mg/dL) with unknown baseline, and serum albumin was 2.2 g/dL (normal: 3.6-5 gm/dL). Erythrocyte sedimentation rate (ESR) was 93 mm/h (normal: 0-20 mm/h), and C-reactive protein (CRP) was 5.3 mg/L. Urinalysis showed hematuria and proteinuria with microscopic analysis of 41-50 RBCs/HPF and 600 mg/dL protein. A 24-hour urine protein measurement was 2235 mg/24 hours (normal: <150 mg/24 hours), while the urine protein/creatinine ratio was 3.35 mg/mg (<0.2 mg/mg), and the microalbumin/creatinine ratio was 4000 mg/g (normal 0-29 mg/g), as mentioned in Table 1.

**Table 1:** Laboratory values at hospital presentation ANCA: Antineutrophil cytoplasmic antibody; BNP: Brain natriuretic peptide; CRP: C-reactive protein; ESR: Erythrocyte sedimentation rate; HbA1c: Glycated hemoglobin; HPF: High-power field; RBC: Red blood cell

Laboratory test	Patient value	Reference range	Other results
Serum troponin	300 ng/mL	0-18 ng/mL	-
Serum creatinine	5.6 mg/dL	0.6-1.4 mg/dL	-
Albumin	2.2 gm/dL	3.6-5 gm/dL	-
Urine RBC	41-50 RBCs/HPF	0-3 RBCs/HPF	-
Urine protein	600 mg/dL	Negative	-
Urine protein/creatinine	3.35 mg/mg	<0.2 mg/mg	-
Urine microalbumin/creatinine	4000 mg/g	0-29 mg/g	-
24-hour urine protein	2235 mg/24 hours	<150 mg/24 hours	-
BNP	954 pg/mL	0-99 pg/mL	-
ESR	93 mm/h	0-20 mm/h	-
CRP	5.3 mg/L	0-1 mg/L	-
Cryoglobulins	-	-	Negative
Hepatitis B	-	-	Negative
Hepatitis C	-	-	Negative
ANCA	-	-	Negative
Anti-glomerular basement membrane antibody	-	-	Negative
Serum protein electrophoresis	-	-	Negative for monoclonal proteins, C3 and C4
HbA1c	5.9%	4%-5.6%	-

Physical examination revealed a 2/6 pansystolic murmur heard best at the apex radiating to the axilla, along with left ventricular systolic dysfunction and dilation, which supports the diagnosis of secondary (functional) mitral regurgitation due to non-ischemic cardiomyopathy. Bibasilar crackles were auscultated in both lungs, consistent with pulmonary edema, while elevated jugular venous pressure indicated raised right-sided heart pressures and systemic venous congestion. Additionally, she demonstrated pitting edema involving the sacrum and both upper and lower extremities with generalized anasarca.

Her ECG showed sinus rhythm without ST-T wave changes, suggesting the absence of acute ischemia or significant arrhythmias at presentation. Specifically, the lack of voltage criteria for left ventricular hypertrophy (LVH), such as increased R-wave amplitude in leads V5 or V6 and deep S waves in V1 or V2, helped identify hypertensive heart disease or structural cardiac changes. Additionally, there were no notable signs of strain patterns, including ST-segment depressions or T-wave inversions in the lateral leads, which would indicate ventricular repolarization abnormalities often associated with advanced LVH and myocardial fibrosis. The absence of these findings may argue against severe hypertensive cardiomyopathy but does not entirely exclude early or subclinical disease.

**Figure 1:**
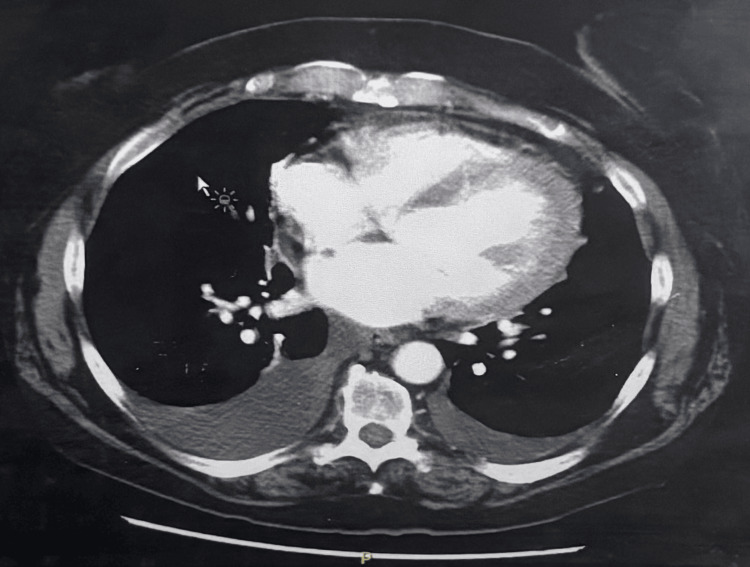
CT scan of the chest showing bilateral pleural effusions, right greater than left, and trace pericardial effusion

The echocardiogram showed a left ventricular ejection fraction (LVEF) of 25%-30%, consistent with moderate to severe systolic dysfunction. Global hypokinesis, without focal regional wall motion abnormalities, was noted, suggesting a non-ischemic cardiomyopathy. The left ventricle was dilated, with mild left atrial enlargement. Evidence of Grade I diastolic dysfunction was present. The chest radiograph confirmed pulmonary edema and bilateral pleural effusions, further corroborating the diagnosis of congestive heart failure with volume overload. As shown in Figure 1, CT scan of the chest showed bilateral pleural effusions, right greater than left, and trace pericardial effusion.

Renal ultrasound showed no atrophic changes, indicating preserved renal structure without chronic damage that could contribute to fluid retention. Further studies were negative for antinuclear antibody, cryoglobulins, hepatitis B, hepatitis C, antineutrophil cytoplasmic antibody (ANCA), anti-glomerular basement membrane, and glycated hemoglobin of 5.9. However, serum protein electrophoresis was negative for monoclonal proteins and C3 and C4, as mentioned in Table 1.

**Figure 2:**
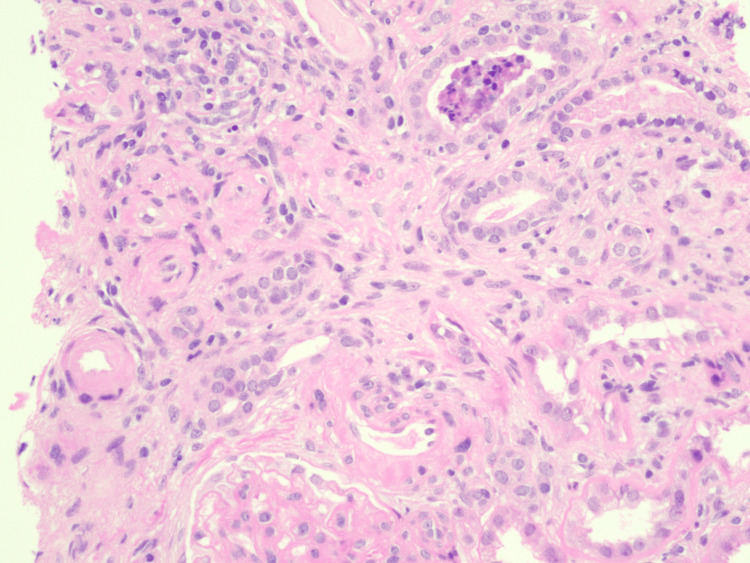
Light microscopy of kidney biopsy with H&E staining 20x magnification view of arteriosclerosis with tubular atrophy and interstitial fibrosis H&E: Hematoxylin and eosin

**Figure 3:**
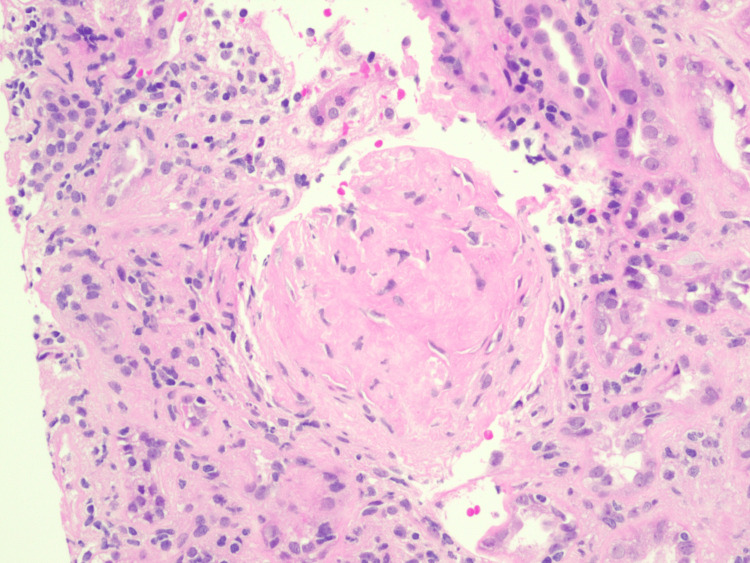
Light microscopy of kidney biopsy with H&E staining 20x magnification view of glomerulosclerosis with fibrocellular crescents H&E: Hematoxylin and eosin

The FGN diagnosis was based on nephrotic-range proteinuria, elevated BNP, and renal biopsy findings. Approximately 14 glomeruli were present for evaluation of renal biopsy, and seven of them (50%) demonstrated fibrocellular crescents with underlying segmental sclerotic lesions, indicating a significant degree of chronic glomerular injury. Staining patterns (IgG, C3, kappa, and lambda) suggest polyclonal deposits, suggesting a non-monoclonal process. Congo red stain was negative, excluding amyloidosis and differentiating it from other fibrillary disorders. Figures 2, 3 show severe arteriosclerosis with tubular atrophy, interstitial fibrosis, and glomerulosclerosis with fibrocellular crescents. Electron microscopy was consistent with the expansion of mesangial regions, non-branching, randomly oriented fibrils typical for FGN, with widespread effacement of podocyte foot processes.

Immunofluorescence studies showed linear staining along tubular and glomerular basement membranes with albumin and diffuse global smudgy staining along glomerular basement membranes and in the mesangial regions with IgG (3+), IgM (trace), C3 (3+), kappa (3+), and lambda light chains (3+), and the diagnosis of FGN was confirmed by an immunohistochemical stain for DNAJB9 alongside a negative Congo red stain.

Her clinical condition was thought to be complicated by left ventricular dysfunction, most likely due to a combination of volume overload, hypertension, and cardiomyopathy secondary to FGN. The patient was initially treated with IV furosemide (Lasix) 40 mg twice daily (BID) for volume overload. Due to persistent signs of congestion (elevated jugular venous pressure, bibasilar crackles, and peripheral edema), the dose was escalated to 80 mg IV BID within 48 hours. The total IV furosemide dose over the first 72 hours was 480 mg. Urine output increased to an average of 2.5-3 L/day after dose escalation, with a cumulative net negative fluid balance of approximately 4.2 L over 72 hours. The patient experienced a weight loss of 3.1 kg (6.8 pounds) from the admission baseline. Nitroglycerin infusion was initiated at 10 mcg/min and titrated to 20 mcg/min for BP control and preload reduction. During hospitalization, BP gradually decreased with IV nitrates and diuretics. By day three, systolic BP lowered to ~140-150 mmHg, allowing initiation of beta-blocker and adjunct antihypertensive therapies such as carvedilol (Coreg) at 3.125 mg BID to reduce afterload and provide beta blockade in left ventricular dysfunction. 

Despite treatment, the patient's kidney dysfunction progressed, leading to acute tubular necrosis due to volume overload, which required dialysis and indicated severe renal compromise. Treatment was initiated after a multidisciplinary discussion involving nephrology and hematology, with pathology review confirming non-monoclonal and polyclonal FGN. Prednisone was initiated at 1 mg/kg/day (typically 60 mg daily) and maintained for eight weeks, followed by a slow taper over 10 months. Final discontinuation was made at month 12. In addition, rituximab 375 mg/m² IV weekly × 4 doses (standard induction course) was administered in coordination with hematology to minimize immunologic flare. 

Her creatinine improved from 5.6 to 3.2 mg/dL, with a decrease in nephrotic range proteinuria to less than 1.3 g/dL at 12 months, requiring less frequent hemodialysis. Inflammatory markers such as CRP and ESR normalized alongside BNP and troponin. Her LVEF improved to 55%-60% at 6 months, mitral regurgitation murmur resolved at 12 months. Nuclear stress testing at the 6-month follow-up showed routine perfusion imaging, no wall motion abnormality, and a normal ejection fraction, as shown in Tables 2-4, confirming the reversibility of cardiac involvement in response to immunosuppression therapy.

**Table 2:** Systemic markers over time CRP: C-reactive protein; ESR: Erythrocyte sedimentation rate

Time point	CRP	ESR	Interpretation
Baseline	Elevated	Elevated	Systemic inflammation present
12 months	Normalized	Normalized	Systemic inflammation controlled

**Table 3:** Renal response over 12 months

Time point	Serum creatinine (mg/dL)	Proteinuria (g/day)	Dialysis frequency
Baseline	5.6	2.2	3 sessions/week
3 months	4.2	1.9	Reduced to 2 sessions/week
6 months	3.6	1.5	1 session/week
12 months	3.2	1.3	Occasional PRN dialysis (1–2x/month)

**Table 4:** Cardiac recovery metrics over time BNP: Brain natriuretic peptide; LVEF: Left ventricular ejection fraction; N/A: Not applicable

Metric	Day 1 (admission)	Day 3	Month 3	Month 6	Month 12
BNP	954 pg/mL	400 pg/mL	N/A	Normalized	N/A
High-sensitivity troponin	300 ng/L	18 ng/L	N/A	Normalized	N/A
LVEF (echocardiogram)	N/A	N/A	N/A	Normalized (55%-60%)	55%–60%
Nuclear stress test findings	N/A	N/A	N/A	Normal perfusion, no wall motion abnormality with normal ejection fraction	N/A
Mitral regurgitation murmur	Present	N/A	N/A	N/A	Resolved

## Discussion

FGN mimics amyloidosis with fibrillary deposits but is distinguished by negative Congo red staining (~15-25 nm) and markers such as DNAJB9 [1]. FGN shows mesangial expansion with IgG, C3, and light chains, whereas amyloidosis features Congo red-positive, extracellular fibrils (7-15 nm) with apple-green birefringence [1,2]. Light chain deposition disease (LCDD) presents with granular, non-fibrillar deposits, often monotypic κ chains. Ultrastructurally, FGN fibrils are ~20 nm and randomly oriented, amyloid fibrils are smaller and β-pleated, while LCDD deposits are non-fibrillar [3,4]. FGN differs from immune complex cardiomyopathies, which involve inflammation; FGN causes direct fibril buildup leading to heart and kidney damage, often causing failure. Unlike typical cardiac-renal syndromes, FGN's pathology involves fibril deposits with minimal inflammation. Immune complex cardiomyopathies result from circulating immune complexes causing myocarditis, dilation, and heart failure, and are treated with immunosuppressants. Recognizing these differences is key for diagnosis and targeted treatment [3-6]. 

Diagnosis of FGN-related cardiomyopathy should start promptly with renal and cardiac biopsies, along with immunohistochemical analysis and electron microscopy of immunoglobulin deposits in the glomeruli and myocardium [4-6]. In our case, severe hypertension and water retention caused by nephrotic syndrome indirectly contributed to cardiomyopathy. FGN cardiomyopathy can often pose a significant diagnostic challenge due to limited evidence of underlying pathophysiology and a lack of diagnostic tools, making targeted therapies challenging to develop. Since cardiac involvement is rare, biopsies are mainly for cases where non-invasive imaging and clinical evaluation cannot distinguish FGN-related cardiomyopathy from other causes. Histological confirmation, including the detection of fibrillary deposits and immunohistochemical markers (such as DNAJB9 in renal FGN, although cardiac-specific markers are less established), can aid in diagnosis [7]. Initial diagnosis favors non-invasive methods such as cardiac MRI and PET scans to assess tissue characteristics and inflammation, reserving endomyocardial biopsy for inconclusive cases due to its procedural risks, such as cardiac perforation, arrhythmia, bleeding, and valvular damage [6,7].

Treatment of kidney dysfunction in FGN-induced cardiomyopathy focuses on controlling inflammation and proteinuria with corticosteroids and supportive care, including angiotensin-converting enzyme inhibitors or angiotensin II receptor blockers, and a low-sodium diet. Severe or resistant cases may require immunosuppressants such as cyclophosphamide, rituximab, mycophenolate mofetil, tacrolimus, or calcineurin inhibitors such as cyclosporine to be used cautiously [7,8]. Since FGN often coexists with cardiomyopathy, standard heart failure treatments and dietary measures are also crucial. Regular monitoring of kidney and heart function guides therapy adjustments [8,9]. Prednisone monotherapy in FGN demonstrates variable and often limited efficacy compared to its use in other forms of glomerulonephritis. Response rates to prednisone alone are generally low, with many patients failing to achieve significant or sustained reduction in proteinuria or stabilization of kidney function. Clinical studies have shown that steroid monotherapy rarely induces remission, and improvements are often modest or transient. This limited effectiveness is attributed to the complex immune-mediated pathogenesis of FGN and the often-advanced disease stage at diagnosis. Consequently, prednisone is frequently used in combination with other immunosuppressive agents to enhance treatment response, especially in patients with more severe or progressive disease [7-10].

The reversibility of cardiomyopathy associated with FGN remains poorly documented, and current treatments are experimental or extrapolated from other immune-related conditions [8-10]. Obinutuzumab is under investigation for FGN, but its safety and efficacy are unproven. Similarly, eculizumab, a monoclonal antibody targeting complement C5, shows potential in complement-mediated kidney diseases but is experimental in FGN, with mixed retrospective outcomes and no definitive evidence of benefit. Stem cell therapy for FGN-related cardiomyopathy is highly speculative, lacking direct preclinical or clinical data. While stem cells have shown promise in other cardiomyopathies, specific studies for FGN are absent. Patients should participate in clinical trials or seek specialized consultation for personalized treatment options [9,10].

Patients with ESRD may benefit from renal transplantation, but recurrence of FGN occurs in 21%-36% of cases, often around 10 years after transplant [8-10]. Younger recipients are at higher risk, while pretransplant monoclonal gammopathy does not predict recurrence. Recurrence is usually detected via protocol biopsies and may be asymptomatic. Long-term monitoring, including DNAJB9 staining, is crucial for early detection. Biopsy decisions should be individualized, reserved for specific clinical indications, rather than routine screening [6-10]. 

Delayed diagnosis of cardiomyopathy in FGN adds a layer of complexity, with a more severe form of the disease leading to the risk of additional organ dysfunction (e.g., the liver, spleen, and gastrointestinal tract) and significant mortality [6-10]. It is crucial to maintain vigilant follow-up of these patients to assess disease activity, recurrence, and potential complications of infections from immunosuppressive drugs by regularly measuring cardiac and renal function. Routine renal biopsies are unnecessary [7-10]. Repeat biopsy should be individualized based on clinical judgment, supported by serologic and urine markers, and mainly employed when results will directly impact management decisions [8-10]. It is reserved for specific situations such as diagnostic uncertainty, unexplained worsening of kidney function, persistent or worsening proteinuria, new or recurrent symptoms, non-responsiveness to therapy, or when guidance on immunosuppressive escalation is needed [6,7-10].

FGN prognosis worsens with male sex, crescents, and renal impairment, which influence treatment and outcomes. Low estimated glomerular filtration rate (<45 mL/min/1.73 m²) and high serum creatinine predict rapid progression to end-stage kidney disease (ESKD), highlighting the need for early detection and targeted therapy. Recognizing these markers guides biopsy, treatment, and trial eligibility [8,9]. Despite current therapies, these factors indicate higher ESKD risk, with immunosuppressants such as rituximab offering some benefit. Early, personalized intervention and research efforts are crucial for improving prognosis [8-10].

## Conclusions

FGN primarily affects the kidneys but can rarely present with extra-renal manifestations, including cardiac involvement. Although data on the reversibility of cardiac dysfunction in FGN-related cases remain limited, early identification and intervention are crucial, as timely treatment may help reverse or mitigate cardiac impairment. A multidisciplinary management approach is essential to optimize patient outcomes. This collaborative strategy enables comprehensive care addressing both renal and extra-renal manifestations, promoting early diagnosis, individualized treatment, and vigilant monitoring to improve prognosis in patients with FGN.
